# Reviving the “Moore Swab”: a Classic Environmental Surveillance Tool Involving Filtration of Flowing Surface Water and Sewage Water To Recover Typhoidal *Salmonella* Bacteria

**DOI:** 10.1128/AEM.00060-20

**Published:** 2020-06-17

**Authors:** Michael J. Sikorski, Myron M. Levine

**Affiliations:** a Center for Vaccine Development and Global Health, University of Maryland School of Medicine, Baltimore, Maryland, USA; b The Institute of Genomic Sciences, University of Maryland School of Medicine, Baltimore, Maryland, USA; c Department of Microbiology and Immunology, University of Maryland School of Medicine, Baltimore, Maryland, USA; d Samoa Typhoid Fever Control Program, Ministry of Health, Apia, Samoa; e Department of Medicine, University of Maryland School of Medicine, Baltimore, Maryland, USA; f Department of Pediatrics, University of Maryland School of Medicine, Baltimore, Maryland, USA; Centers for Disease Control and Prevention

**Keywords:** Moore swab, environmental surveillance, typhoidal *Salmonella*, waterborne pathogens, environmental bacteriology, filtration

## Abstract

The “Moore swab” is a classic environmental surveillance tool whereby a gauze pad tied with string is suspended in flowing water or wastewater contaminated with human feces and harboring enteric pathogens that pose a human health threat. In contrast to single volume “grab” samples, Moore swabs act as continuous filters to “trap” microorganisms, which are subsequently isolated and confirmed using appropriate laboratory methods. Continuous filtration is valuable for the isolation of transiently present pathogens such as human-restricted Salmonella enterica serovars Typhi and Paratyphi A and B. The technique was first proposed (1948) to trace *Salmonella* Paratyphi B systematically through sewers to pinpoint the residence of a chronic carrier responsible for sporadic outbreaks of paratyphoid fever. From 1948 to 1986, Moore swabs proved instrumental to identify long-term human reservoirs (chronic carriers) and long-cycle environmental transmission pathways of *S*. Typhi and Paratyphi, for example, to decipher endemic transmission in Santiago, Chile, during the 1980s. Despite limitations such as intermittent shedding of typhoidal *Salmonella* by humans and the effects of dilution, *S*. Typhi and *S*. Paratyphi have been recovered from sewers, surface waters, irrigation canals, storm drains, flush toilets, and septic tanks by using Moore swabs. Driven by the emergence of multiple antibiotic-resistant *S*. Typhi and *S*. Paratyphi A strains that limit treatment options, several countries are embarking on accelerated typhoid control programs using vaccines and environmental interventions. Moore swabs, which are regaining appreciation as important components of the public health/environmental microbiology toolbox, can enhance environmental surveillance for typhoidal *Salmonella*, thereby contributing to the control of typhoid fever.

## Abstract (precis)

The “Moore swab” is a classic environmental surveillance tool whereby a gauze pad tied with string is suspended in flowing water or wastewater contaminated with human feces and harboring enteric pathogens that pose a human health threat. In contrast to single volume “grab” samples, Moore swabs act as continuous filters to “trap” microorganisms, which are subsequently isolated and confirmed using appropriate laboratory methods. Continuous filtration is valuable for the isolation of transiently present pathogens such as human-restricted Salmonella enterica serovars Typhi and Paratyphi A and B.

## INTRODUCTION

**Looking at the fact in all its bearings, the only rational explanation of it seems to be, that the sewer does not propagate intestinal fever because it exhales foul gases, but solely because it is the actual recipient and vehicle of the fever poison.**

—William Budd (1)

William Budd’s incrimination of sewage effluents as a major contaminant and cause of enteric illness (1) preceded Gaffky’s isolation of Salmonella enterica subspecies *enterica* serovar Typhi as the pathogenic cause of typhoid fever in 1886 (2) by 25 years. Recovery of *S*. Typhi from sewage became routine after the introduction of highly selective Wilson and Blair’s bismuth sulfite agar in 1927 (3), which allowed isolation of *S*. Typhi from complex mixed sewage and fecal samples (4–9). Indeed, since these discoveries, wastewater has been repeatedly subject to systematic surveillance, and outbreak responses to enteric illnesses have relied upon practical methods to detect pathogens in sewage and contaminated water.

**FIG 1:**
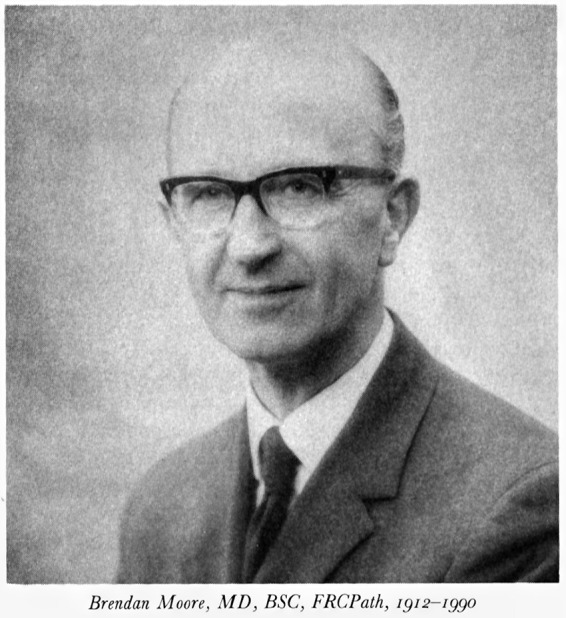
Portrait of Brendan Moore. (Reprinted with permission from the *Report and Transactions of the Devonshire Association for the Advancement of Science* [133].)

In 1946, Brendan Moore (Fig. 1) of the Public Health Laboratory of Exeter was called upon to identify the sources of infection responsible for clusters of paratyphoid fever cases in North Devon, England, between 1943 and 1945 and for a larger outbreak of 25 cases in 1946 (10). Moore’s epidemiologic investigation linked the cases to a swimming area likely contaminated with sewage carrying Salmonella enterica subspecies *enterica* serovar Paratyphi B, the agent of paratyphoid B fever, and with consumption of ice cream from a truck vendor (10). Previously, environmental sources that had been shown to be contaminated with paratyphoid bacilli included untreated sewage (9) and feces-contaminated drinking/cooking water (11). For decades, public health officers had utilized sewage surveillance to detect shedders of these bacilli by inoculating sewage samples into enrichment broths, subculturing the broth onto selective media, identifying suspicious colonies, further subculturing to confirm characteristic biochemical profiles, serotyping by agglutination with specific antisera to surface antigens, and, when relevant and available, further typing with bacteriophages (12).

To identify the specific foci of infection behind these North Devon outbreaks, Moore and colleagues applied systematic “sewage tracing” (10, 13). Moore’s predecessors would have expended enormous human and material resources to collect directly a volume of sewage at single time points, i.e., “catch” or “grab” sampling, across the town and then to check each sample for evidence of paratyphoid bacilli, while hoping that a sampling event would coincide with fecal excretion of paratyphoid bacilli into one of the sewers. Moreover, fecal excretion of *S*. Typhi and *S*. Paratyphi by short-term or chronic carriers may be intermittent (14–17), rendering even aggregates (composites) of several grab samples over an interval inadequate. To preserve resources and overcome the challenge posed by intermittent shedding, Moore sought to lay “traps” in the municipal sewers that could filter the microbial contents of flowing sewage continuously over extended periods of time.

A simple technique was therefore used of taking a piece of gauze about four feet in length and six inches wide, folding it into a pad of eight thicknesses and attaching it firmly by one end to a long piece of stout string. The gauze was then immersed in the flowing sewage, the string attached suitably just under the manhole cover, and the gauze left in position for 48 hours (10).

Moore applied the same classical bacteriology techniques of his day to enrich, select, isolate, and confirm *S*. Paratyphi B from these integrated sewer samples. He traced excretion of paratyphoid B bacilli to the single-family home of the local ice cream truck vendor and his wife. Fecal cultures from the ice cream truck owner’s wife confirmed that she was a chronic (putative gallbladder) carrier of paratyphoid bacilli and the likely source of infection for the 1946 outbreak. In North Devon, and in Sidmouth, Moore refined his technique for tracing excretion of *S*. Paratyphi and *S*. Typhi to single blocks and housing units (13, 18, 19).

This humble innovation to traditional sewage sampling paved the way for dozens of identifications of residual excreters of *Salmonella* Typhi and Paratyphi over the subsequent decades. Named after its dutiful inventor, this device became known as the “Moore swab” or generically as the “sewer swab,” “gauze pad,” or “sewer pad” method of trap sampling for enteric pathogens in sewage. Over the past 75 years, Moore’s classical technique for the detection of typhoidal *Salmonella* in sewage has been adapted to many fecal borne pathogens, including Vibrio cholerae O1 (20–26), *Aeromonas* spp. (27, 28), nontyphoidal *Salmonella* serovars (29), Escherichia coli O157:H7 (30), *Campylobacter* spp. (31), and poliovirus (32–38), among others (39). The utility of the method has expanded beyond the tracing of active pathogen excretion in sewage and rivers to include investigations of ongoing outbreaks, systematic environmental surveillance, bacterial enumeration in surface waters, and septic tank surveillance. In the ensuing sections, we first provide brief instructions on the Moore swab technique and its construction and then highlight several of these expanded uses, focusing on the detection of typhoidal *Salmonella* and Vibrio cholerae O1 as archetypal examples. We aim to stimulate new public health expertise and revitalize this versatile technique in both academic and public health laboratories where enteric disease surveillance and outbreak response to fecally transmitted human pathogens are integral to control efforts.

## MOORE SWAB CONSTRUCTION AND PARAMETERS

**FIG 2:**
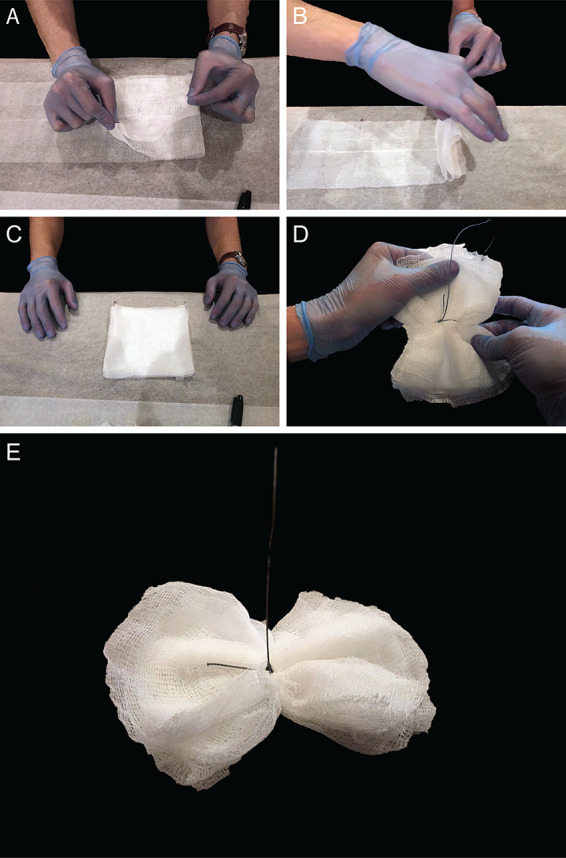
Constructing a Moore swab. (A and B) A length of gauze, 6 inches by 48 inches, is folded onto itself in a pleated pattern to form a pad. (C and D) The gauze pad is tied at the center with high-test fishing line. (E) The Moore swab may be suspended in flowing sewers or surface waters.

Moore swabs are crafted using strips of cotton gauze cut into 6-inch by 48-inch lengths and folded eight times until an 8-ply square pad is formed (Fig. 2). The 6-inch by 6-inch square-pad is tied by a string, twine, wire, or fishing line around the center (10, 18, 40) and often sterilized in an autoclave. For a defined and controllable mesh size, cheese cloth may be substituted in place of cotton gauze (41–44). Long slits may also be cut into the square pad (41) or the uncut pad may simply be tied at a corner. Variations of the Moore swab have rendered it suitable for sampling typhoidal *Salmonella* from water closets (45), poliomyelitis virus from sewers (38), and other enteric pathogens from surface waters (46).

After its introduction, the method was expanded upon with variations on the setting, swab parameters, and postsampling enrichment and selection methods (Table 1). In recognition of differences in preference and available resources among enteric laboratories globally, the parameters and bacteriological steps in Table 1 represent examples drawn from the literature and author experience.

**TABLE 1:** Method considerations and parameters for the isolation and identification of *S*. Typhi using the Moore swab technique

Step	Options	Details or examples (references)
Site/source selection	Untreated effluent	Public sewer network (10, 18, 40, 56, 60, 61, 63); sewage leaving a building, residence, or hospital (55, 62, 64, 92); repurposed wastewater, e.g., for irrigation (69, 76)
	Treatment plant influent/effluent	Wastewater entering/exiting a treatment plant (134, 135)
	Environmental	Surface water, e.g., canals, rivers, streams, and storm drains (55, 57, 58, 76)
	Flush toilets	Toilet prior to sewer (45) or septic tank (126)
Parameters	Swab construction	Gauze type (fiber composition, mesh size, absorbency), gauze measurements (by size or by mass), string or wire attachment
	Sampling schedule	Frequency, duration, replicates
	Sample handling and processing	Transport conditions and time
	Dilution schema	Direct inoculation, 5- or 10-fold dilution schema
Enrichment	Preenrichment	Universal preenrichment broth, nutrient broth, sterile saline
	Selective enrichment	Varies by species, e.g., selenite-F broth for *Salmonella*
Differentiation/selection	Differential solid media	For enteric bacteria, e.g., *Salmonella-Shigella* agar, deoxycholate-citrate agar, xylose-lysine-deoxycholate agar
	Highly selective solid media	Varies by species, e.g., Wilson and Blair (bismuth sulfite) agar for *S*. Typhi
Identification (classical)	Biochemical reactions	Triple sugar iron agar, lysine iron agar, enzyme activity, urea broth, fermentation, commercial kits/panels
	Serotyping	For *Salmonella*, agglutination with anti-O, anti-H, and anti-Vi antisera
Identification (modern)	Pulse-field gel electrophoresis	
	PCR	PCR or quantitative real-time PCR
	Genomics	Whole-genome, 16S ribosomal, or shotgun sequencing

## *SALMONELLA* TYPHI AND PARATYPHI

Typhoid and paratyphoid fevers, caused by ingestion of *S*. Typhi and *S*. Paratyphi A or B, respectively, are enteric illnesses that emerged as ideal candidates for environmental surveillance based on their epidemiology. These human host-restricted infections (47) by definition have no animal reservoir and are spread from person to person via the fecal-oral route by the consumption of food or water vehicles contaminated with the pathogen, either directly through short-cycle or indirectly through long-cycle transmission. The former usually takes place through a food vehicle contaminated by a temporary or chronically (≥12 months) infected individual who is shedding bacteria in their feces. Asymptomatic chronic carriers can harbor *S*. Typhi or *S*. Paratyphi in their gallbladders and intermittently excrete bacteria for decades. In long-cycle transmission, humans become infected from the consumption of vehicles derived from environmental contamination such as untreated sewage/septic system effluents reaching untreated water supplies or being used to irrigate crops that are consumed uncooked (48, 49). Viable *S*. Typhi bacilli have been recovered from septic tanks after 24 to 27 days (50, 51), vegetables after 1 to 2 months, and soil after 2 to 3 months (52, 53). *S*. Typhi can also travel considerable distances through soil, groundwater, and surface water (54). Thus, surveillance for these pathways is key to interrupt amplified transmission in high-incidence populations and to detect and eliminate residual reservoirs (i.e., temporary and chronic carriers).

Historically, it had been difficult to grow typhoidal *Salmonella* serovars from environmental sources; however, 20th century milestones in the formulation of enrichment broths, selective media, and both serotyping and bacteriophage typing markedly expanded the toolbox of the public health and environmental officer (12). After Moore’s classical publications, the technique was rapidly adopted across the United Kingdom and the United States to solve similar challenges—sporadic but persistent outbreaks of typhoid fever in small towns—epidemiologically akin to Moore’s experiences in North Devon and Sidmouth. Each group expanded upon Moore’s initial methodology to suit the needs of their surveillance program and to compare bacteriological methods.

### Tracing a chronic carrier through sewers and rivers.

The classical use of the Moore swab, as described, was to trace an unknown but suspected chronic carrier through sewer or river sampling. The dilemma was typical: a small outbreak of a few cases of enteric fever would occur every few years or so, oftentimes of a single phage type, and a public health official or medical officer would eventually suspect an asymptomatic chronic gallbladder carrier of typhoidal *Salmonella* in the town. Moore swabs were systematically deployed in the jurisdiction at select sewage access sites and rivers or streams epidemiologically linked to the cases. Moore’s initial approach as described in 1950:

The infected swab, collected after 48 hours from the manhole under examination, is delivered to the laboratory in a sterile container. It is washed with 20 ml of nutrient broth and the washings pipetted off into a sterile tube. Three 5-fold dilutions of the infected material are plated in 0.1 ml volumes on Wilson and Blair’s medium, the latter plates being carefully dried before incubation. Five milliliters of washings are inoculated into 200 ml of selenite medium in a liter flask. After overnight incubation, the selenite medium is subcultured (a) to Wilson and Blair’s medium for typhoid colonies, and (b) to ‘mannitol-lead-acetate’ medium for other organisms of the *Salmonella* group (13).

Moore confirmed suspicious colonies by biochemical and agglutination methods.

When typhoidal *Salmonella*-positive points were discovered and mapped, additional swabs were deployed upstream of sewage flow of the positive point. This was repeated systematically until results showed that the swabs were positive downstream and negative upstream of an identifiable point of possible contamination, such as a block of houses sharing a common sewer (55). This general approach was also used in sampling rivers and streams until a pipe carrying wastewater with human fecal material was identified (56, 57) or septic tanks were observed to be located nearby (58). In all cases, a team of public health workers was then required to interview the people living at or near the suspected source and to obtain stool samples to confirm bacteriologically the silent excretion. Confirmation of the typhoidal *Salmonella* serovar and bacteriophage type further supported links between clinical isolates and isolates recovered from the environment. Frequently, chronic carriers discovered by this method gave no past medical history of enteric illness, emphasizing the importance of unbiased surveillance approaches. Finally, the utmost care for confidentiality was maintained during these investigations to avoid the social stigmatization of someone being labeled as a chronic typhoid carrier (59).

In the town of Purbrook, United Kingdom (population, ∼6,000), five pediatric cases of typhoid that occurred over 4 years led public health authorities to collect grab samples of water from the local river downstream of the town. One grab sample in 1949 finally yielded *S*. Typhi. Lendon and Mackenzie (56) observed that an overflow sewage pipe discharged into the river where the grab sample had been positive for *S*. Typhi. Accessing manholes, they systematically placed Moore swabs at sewer junctions upflow along the sewage pipe, documenting positives until they reached a point where the swabs became negative. They found the sewage pipe from a single household to be positive and this proved to be the home of a 70-year-old chronic carrier of *S*. Typhi.

In Farnham, United Kingdom, Hobbs (60) reported on a case of typhoid in a 7-year-old child who had exposure to a sewage-contaminated river and the use of Moore swabs to trace and pinpoint the house of a carrier. Greenberg et al. (40) and Shearer et al. (61) described detection of a single carrier in the isolated town of Portola, CA (population, ∼2,200), via use of Moore swabs in sewers; that carrier had been responsible for cases of typhoid occurring intermittently over ∼5 years. Robinson (62) used the sewer of a mental health facility housing known carriers of *S*. Typhi and *S*. Paratyphi as a site to compare methods: gauze versus alginate wool swabs, five different liquid enrichment media, four solid selective media, and dilution techniques. His conclusions were that selenite-F broth was most effective for enrichment of *S*. Typhi from sewage, that Wilson and Blair agar was the most selective medium, outperforming MacConkey agar, and that dilutions offered variable results likely dependent on sewage composition but should be included. In British Columbia, Bowmer et al. (58) were first to trace a chronic typhoid carrier household via storm drains and septic tank outflows. Interestingly, two phage types were present in the carrier, but only one was recovered from the infected children who had played downstream in the contaminated water. Pilsworth (63) traced a typhoid carrier through sewage in West Mersea, United Kingdom, and affirmed that 37°C was ideal for incubations. In 1964, Bokkenheuser (14) authored a comprehensive review of methodology to detect typhoid carriers. These classic investigations are listed in Table 2, with notable variations and unique perspectives discussed in ensuing sections.

**TABLE 2:** A chronological review of the classical uses of Moore swabs and methods thereof for the filtration of typhoidal *Salmonella* from environmental samples^*a*^

Author(s)	Publication yr	Organism(s)	Prompting circumstances	Location(s)	Sampling site(s)	Date(s)	Time *in situ*	Bacteriological methods^*b*^	Reference
Moore	1948	*S*. Paratyphi B	A paratyphoid outbreak in a small coast town from June–August 1946 yielded 25 diagnosed cases of paratyphoid fever and preceded by sporadic cases: two in 1943, three in 1944, and six in 1945. Sewer samples remained positive for paratyphoid B bacilli for 18 mo after	North Devon, UK	Municipal sewers (25 manholes)	1946–1948	48 h	Selenite-F broth, desoxycholate-citrate medium, Vi-bacteriophage typing	10
Moore	1950	*S*. Typhi	Modification of bacteriological methods to detect *Salmonella* Typhi in sewers and a description of various comparative experiments on the relative efficiency of standard bacteriology methods	North Devon, UK	Municipal sewers	Not reported	48 h	Nutrient broth, Wilson and Blair agar, selenite enrichment broth, mannitol-lead-acetate medium, lactose-sucrose medium, agglutination, biochemical and serological testing, Vi-bacteriophage typing	13
Cruickshank	1950		Review of Vi antibody tests, culture media, systematic sewer tracing, and Vi-bacteriophage typing for their utility and importance						19
Jones	1951	*S*. Typhi	A sporadic case of typhoid fever in a hospital engineer who cleaned the hospital sewer was diagnosed 14 mo after a massive hospital outbreak involving 125 cases	Oswestry, UK	Hospital sewer: Main and six traps	28 July 1949 to 13 March 1950	48 h	Selenite-F broth, deoxycholate citrate agar, Wilson and Blair agar	64
Lendon and Mackenzie	1951	*S*. Typhi	Two cases of typhoid fever in 1944 and three cases in 1948 appeared independently associated with a particular river located next to a sewer overflow	Purbrook, UK	Municipal sewers	1948–1949	5–7 days	Selenite-F broth, deoxycholate citrate agar, Wilson and Blair agar	56
Moore, Perry, and Chard	1952	*S*. Typhi, Paratyphi	1–4 cases occurring every 1–4 years since 1930, suggestive of a local human reservoir of infection	Sidmouth, UK	Municipal sewers	1946–1949	48 h	Nutrient broth, selenite-F broth, Wilson and Blair agar, Hynes’ deoxycholate-citrate medium, agglutination, biochemical and serological testing, Vi-bacteriophage typing	18
Kwantes and Speedy	1955	*S*. Paratyphi	Outbreaks of paratyphoid fever occurred in 17 people in 1952 and 5 people in 1953, prompting suspicions of a paratyphoid carrier	Goodwick, Wales, UK	Water Closet (Direct toilet)	1954	3–4 days	Selenite-F broth, Wilson and Blair agar	45
Kelly, Clark, and Coleman	1955	Coxsackie, Mycobacterium tuberculosis, *S*. Typhi	Five cases of typhoid fever occurred over 3 years in families living within 1 mile of a creek downstream of a sewage plant	New York State, USA	Sewage plants, sewers	Not reported	4 days	Buffered 30% glycerol, tetrathionate medium, bismuth sulfite agar, modified Endo's agar, Vi-bacteriophage typing	134
Murdock and Lawson	1955	*S*. Typhi	Several cases of typhoid fever in adolescents—three in 1951, three in 1953, and two in 1954—were of the same Vi-phage type C and seemed linked to a pair of streams	Belfast, Northern Ireland	Stream, fishpond	1954	72 h	Selenite-F broth, bismuth sulfite medium, Vi-bacteriophage typing	57
Hobbs	1956	*S*. Typhi	A 7-year-old boy diagnosed with typhoid fever from unknown source reportedly played near sewage overflow from heavy rains. Five historical cases of typhoid had occurred over the past 28 years	Farnham, UK	Municipal sewers	1955	3 days	Not reported	60
Greenberg, Wickenden, and Lee	1957	*S*. Typhi	A carrier was suspected in four cases of typhoid fever occurring over 5 years	Portola, CA	Municipal sewers	20 February to 1 April 1957	48 h	Selenite-F, bismuth sulfite agar, TSI, urea agar, Vi-bacteriophage typing	40
Shearer, Browne, Gordon, and Hollister	1959	*S*. Typhi							61
Bloom, Mack, and Mallmann	1958	*Salmonella* spp.	Methods were compared to isolate *Salmonella* from sewage before, after, and downstream from sewage plants and pumping stations	Lansing and East Lansing, MI	Sewage plants and pumping stations	October 1956 to December 1957	24–72 h	Tetrathionate broth, selenite brilliant green medium, bismuth sulfite agar, biochemical and serological testing	135
Robinson	1958	*S*. Typhi	Surveillance in a local mental health hospital where known carriers of *S*. Typhi and Paratyphi resided	Not reported	Hospital sewer	Not reported	48 h	5 different liquid media, 4 different solid media	62
Bowmer, Hudson, and Sunderland	1959	*S*. Typhi	Four cases of typhoid fever of the same phage type were diagnosed in 1953, 1955–1957	Burnaby, British Columbia, Canada	Storm drains	18 February to 1 April 1957	48 h	Nutrient broth, bismuth sulfite agar, biochemical and serological testing	58
Pilsworth	1960	*S*. Typhi	20 cases of typhoid fever between August 1950 and December 1956, some of similar phage types	West Mersea, UK	Municipal sewers: 11 drainage areas and 1 pump	November 1952 to May 1954	3 days	Sterile saline, selenite broth, selenite brilliant green broth, deoxycholate citrate agar, Wilson and Blair agar, biochemical and serological testing, Vi-bacteriophage typing	63
Bokkenheuser	1964		Review of Vi antibody tests, chronic carrier state, specimen transportation, enrichment media, isolation media, sewage, and bacteriophage typing for the detection of typhoid carriers						14
Callaghan and Brodie	1968	*S*. Typhi	Laboratory aspects of a large postoutbreak surveillance program	Aberdeen, Scotland	Large-diameter municipal sewers	July 1964 to September 1966	7 days	Ringer solution, mannitol selenite broth, modified bismuth sulfite agar, modified *Salmonella-Shigella* agar, biochemical and serological tests, Vi-bacteriophage typing	136
Moore	1971		Review of history of sanitation and bacteriology practices important in typhoid investigations and control, and discussion of challenges ahead						12
Conn, Heymann, Jamieson, McWilliam, and Scott	1972	*S*. Typhi	Eight cases in Edinburgh from 1963–1970; in summer 1970, three of these directly connected with drinking from the Water of Leith river	Edinburgh, UK	River, draining surface water, household sewage	27 July to 27 October 2019	7 days	Selenite-F broth, deoxycholate citrate agar, MacConkey medium, Kohn's two-tube medium, biochemical and serological tests, phage typing	55
McGinnis and DeWalle	1983		Review of the data available on the movement and survival of typhoid bacilli under various environmental conditions, including soil, septic tanks, sewers, and rivers						54
Sears, Ferreccio, Levine, Cordano, Monreal, Black, Ottone, Rowe, and Chilean Typhoid Committee	1984	*S*. Typhi	Urban endemicity in a modern industrialized metropolitan area where a majority of household sewage is left untreated prior to its use in crop irrigation	Santiago, Chile	Rivers and irrigation canals	January to March 1983	48–72 h	Selenite-F broth, *Salmonella-Shigella* agar, bismuth sulfite agar, deoxycholate-citrate agar, triple sugar iron agar slants	76

a Gray shading indicates review articles.

b Wilson and Blair agar is bismuth sulfite agar and is sometimes modified in modern preparations. The terminology above follows the citation.

### A residual carrier after a major hospital outbreak.

In 1951, an outbreak of 135 known cases of typhoid fever occurred in a hospital in Oswestry, United Kingdom (64). After meticulous collection of serial stool cultures and Vi-agglutination serology tests of 435 staff, 19 asymptomatic excreters of *S*. Typhi were detected along with 15 incubatory carriers who subsequently became clinical cases. Fourteen months after the outbreak was addressed, yet another case occurred in a hospital employee working at the hospital sewage treatment site. By placing Moore swabs in the main sewer and in sewers draining each of six wards, *S*. Typhi was pinpointed to a single ward. The responsible chronic carrier of *S*. Typhi was detected and linked epidemiologically using Vi-phage typing. A. C. Jones, the public health official behind this work, noted, “this type of sewage examination might well be employed as a supplementary postepidemic method of ensuring that an institution has been cleared of chronic carriers” (64).

### The water-closet (toilet) swab.

Kwantes and Speedy reconfigured Moore swabs into string-wound cylinders to allow their placement directly into water closets (i.e., toilets) to detect a paratyphoid carrier (45). In Goodwick, Wales, this modification was necessary because wastewater exiting numerous houses entered a common drain accessed by a single manhole. This sewer configuration precluded the systematic tracing upstream from the manhole to individual houses. By extending toilet swabbing schedules to accommodate the occupants’ weekend bathroom habits, the individual toilet method proved successful in identifying an unknown carrier of *S*. Paratyphi B (45).

### Sewage-contaminated water.

Three young boys in Belfast, Northern Ireland, who had been playing near a polluted stream became acutely ill with typhoid fever in 1951 (57). Six weeks later, a workman near the stream also fell ill with typhoid having the same Vi-phage type as the boys’ isolates. Murdock and Lawson were unable to link these initial cases epidemiologically nor isolate *S*. Typhi from the stream using Moore swabs (57). Three years passed until two more cases occurred that were associated with exposure to the same stream and another nearby stream. *S*. Typhi of the same Vi-phage type was detected using Moore swabs in water of the second stream and from a septic pipe draining sewage from several houses. Upon inquiry and testing fecal specimens from the residents, Murdock and Lawson discovered a single chronic *S*. Typhi carrier (57). A fluorescein color dye test confirmed the connection between the carrier’s septic effluents and the second stream (57).

Bowmer et al. (58) fittingly entitled their 1959 investigation of four sporadic cases of bacteriophage-matched typhoid fever in schoolchildren from 1953 to 1957 as “Typhoid fever: where there’s a case, there’s a carrier.” Indeed, a single carrier was discovered by employing the Moore swab technique in stormwater drains of a municipality that lacked a piped sewerage system and relied exclusively upon household septic tanks. Bowmer’s team traced the contamination with typhoid bacilli to a roadside stormwater drain that serviced three houses containing seven persons. A 59-year-old woman’s coproculture yielded *S*. Typhi of the same bacteriophage type as the four cases. The carrier subsequently underwent cholecystectomy that revealed *S*. Typhi in her bile, gallstones, and gallbladder wall tissue (58), consistent with typhoidal persistence (65–67).

## THE SANTIAGO, CHILE, EXPERIENCE

During the late 19th and early 20th century, treatment of municipal water supplies by chlorination, sand filtration, or both resulted in a precipitous decline in typhoid fever incidence in the major cities of Europe and North America (68). Installation and maintenance of piped sewerage networks also contributed importantly to control typhoid fever. A major exception to this trend was the metropolitan region of Santiago, Chile, where high annual incidence rates of endemic typhoid fever persisted through the 1980s, despite 96% of households having access to treated, bacteriologically monitored, potable water and 70% to 80% having flush toilets to remove human fecal waste (69). In Santiago during that era, typhoid incidence showed a marked summer seasonality and affected all socioeconomic strata (69). Two hypotheses were posed to explain this enigmatic high-incidence endemicity: (i) an unusually high prevalence of chronic biliary carriers of *S*. Typhi who not only served as a large long-term reservoir of infection but also maintained high-incidence short-cycle transmission through contamination of food vehicles in homes, food kiosks, and restaurants, or (ii) an unconventional irrigation practice in which untreated sewage, after it had coursed through the city, was used to irrigate crops grown in fields in the periphery.

In support of the first hypothesis, which was by far the most widely accepted in the 1970s, Santiago was shown to have an exceptionally high prevalence of cholelithiasis among adults (70). Of 1,000 consecutive Santiago residents (796 females and 204 males) undergoing cholecystectomy in seven hospitals in Santiago from July to October 1980 and who had their bile cultured, 38 (3.8%) bile cultures yielded *S*. Typhi and 35 (3.5%) grew *S*. Paratyphi (30 of 35 were Paratyphi B) (71). Levine et al. used those data, along with the prevalence of cholelithiasis in Santiago by age and sex, to estimate the prevalence of chronic typhoid carriers by age and sex (70). The overall carrier prevalence was 694/10^5^ persons >10 years of age. However, among women aged ≥40 years, the prevalence of *S*. Typhi biliary carriers exceeded 2,000/10^5^ (i.e., >2% of the women). This very high estimated prevalence of typhoid carriers, corroborated subsequently by prospective data from case/control studies (72) and prevalence surveys of food handlers that included stool cultures (73, 74), gave credibility to those who argued that chronic carriers were playing the key role in maintaining the high typhoid incidence in Santiago.

Evidence incriminating the role of environmental contamination being responsible for maintaining the high incidence of typhoid in Santiago required active investigation. Careful inspection of the manner in which raw sewage was handled in Santiago and a series of painstaking environmental bacteriology studies utilizing Moore swabs incriminated the use of untreated sewage in the dry summer months to irrigate extensive fields of vegetable crops that were eaten uncooked as the practice that was responsible for maintaining amplified long-cycle transmission. This could not have been achieved without Moore swabs.

In the 1970s and 1980s, neighborhood sewers in Santiago deposited raw untreated sewage into two waterways, including a giant open sewage canal (Zanjon de la Aguada) that traversed the metropolitan region from east to west and to a lesser extent into the Mapocho River that coursed the city from northeast to southwest in a gentle S-like trajectory. In the southwestern part of the city, the Zanjon emptied its sewage into the Mapocho River, which then coursed for several kilometers without additional sewage entering the river in an attempt to allow self-cleansing of the contaminated water. In the western and southwestern parts of the metropolitan region, these waters, which remained untreated, were subsequently used to irrigate lettuce, cabbage, and celery crops in the rainless summers, i.e., vegetables that were consumed uncooked. These vegetables, which were trucked back to the markets and supermarkets of the city for sale, ultimately contaminated food preparation areas of kitchens in households and restaurants (72, 75).

**FIG 3:**
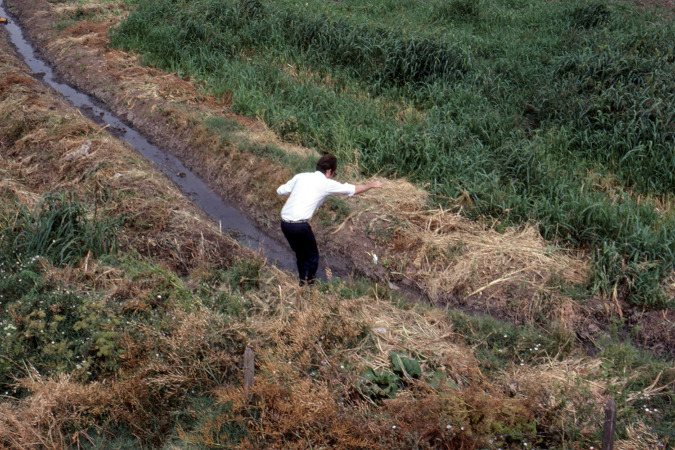
Placement of Moore swabs in the irrigation canals outside Santiago, Chile, in January of 1983.

Moore swabs proved to be a critical tool in elucidating the previously enigmatic mode of transmission responsible for maintaining amplified high-incidence endemic *S*. Typhi disease in Santiago. From January to March, 1983, i.e., during the Chilean summer, Sears and colleagues deployed 133 Moore swabs into irrigation canals (Fig. 3) (76). Of the 93 swabs recovered and cultured, 4 of 45 (8.9%) from the Mapocho River and 4 of 31 (12.9%) from the Zanjon de la Aguada yielded *S*. Typhi. Prior to this, Chilean microbiologists at the Instituto de Salud Pública (Institute of Public Health [ISP]) had used traditional “grab” sampling in their environmental bacteriologic attempts to determine if *S*. Typhi was present in these wastewaters. Notably, only once among 100 grab samples collected over several years was *S*. Typhi isolated (77).

In collaboration with the ISP, Sears et al. used virtually identical bacteriological methods as the ISP had previously employed but substituted the Moore swab method for “grab” sampling (76). This change in collection technique led to repeated recovery of *S*. Typhi from these irrigation waters in 1983, thereby supporting the hypothesis that vegetables irrigated with untreated sewage water were underlying Santiago’s unusual pervasiveness of typhoid endemicity (76). This was also the first demonstration of the efficacy and utility of the Moore swab in recovering typhoid bacilli from large-diameter irrigation canals in a highly endemic urban setting. This observation is highly relevant in sub-Saharan African cities today where urban watercourses function as open sewers.

Four large-scale field trials of live oral typhoid vaccine in ∼500,000 Chilean school children carried out in the 1980s markedly decreased the incidence of typhoid in that high-risk age group (78–81). Based on the evidence generated by Sears and team (76) in collaboration with the ISP, the Ministry of Health recommended to the government of Chile that the use of untreated sewage water to irrigate crops should be prohibited, but this recommendation was not heeded. In 1991, after an absence of a century, cholera returned to South America, resulting in massive outbreaks in Peru (82), Ecuador (83), Argentina (84), and Colombia (85), before spreading more widely through the continent (86). A small outbreak of 41 cases of El Tor cholera hit the metropolitan region of Santiago in April 1991 (87). Facing the threat of cholera, the Ministry of Health and other arms of the government of Chile mobilized rapidly and effectively and finally outlawed the use of untreated sewage water to irrigate crops (75, 88). This ban was strictly policed. Despite the unpopularity of this ban among farmers and agricultural workers whose livelihoods were affected, this intervention abruptly interrupted the cholera outbreak and also led to a precipitous fall in the transmission of typhoid, resulting in the end of amplified endemic transmission (75). Within a few years, only sporadic low-level transmission due to chronic carriers contaminating food vehicles continued, and that residual incidence progressively declined over the following decade. These data confirm the key role that use of contaminated irrigation waters had previously been playing in maintaining amplified transmission and perpetuating endemic typhoid in Santiago (75, 89).

## CHOLERA

Cholera, the diarrheal illness caused by cholera toxin-producing Vibrio cholerae of serogroup O1, in its severe clinical form, cholera gravis, can rapidly dehydrate and kill even healthy adults if water and electrolyte losses are not expeditiously replaced. The current 7th pandemic of cholera due to the El Tor biotype began in 1961 in Southeast Asia, disseminated to south and east Asia during the 1960s, reached Africa by 1970 and South America in 1991. In newly affected areas, cholera tends to occur in explosive epidemics, and in endemic foci, it exhibits marked seasonality. Since the early 1970s, Moore swabs have played an important role in cholera surveillance in threatened as well as cholera-stricken communities and in tracing infected persons with clinical and asymptomatic infections. Examples are described below from low- and middle-income and from high-income countries.

### Anticipatory sentinel sewage surveillance for healthy carriers.

In 1973, widespread occurrence of cholera in neighboring Malawi, Mozambique, and Angola prompted South Africa to initiate surveillance to detect cholera in presumed high-risk populations (20, 21). It was assumed that the many migrant laborers who came from the above-mentioned countries to work long term in South Africa’s coal and gold mines would be likely introducers of V. cholerae O1. Isaäcson et al. undertook a three-pronged surveillance system among 20,000 miners that included (i) systematic Moore swab surveillance of sewers serving the mines and an acclimatization center (21), (ii) expeditious collection of rectal swabs from miners served by sewers that yielded V. cholerae O1 from surveillance Moore swabs and (iii) physical isolation and rectal swab cultures from cases of diarrheal illness to confirm cholera cases.

Although sewer surveillance using Moore swabs began in November 1973, there were no isolations of V. cholerae O1 until late March 1974. Rectal swabs from miners whose toilets were served by the culture-positive sewer line revealed two miners with subclinical infection. One week later, as sewer swabs remained positive, there were six subclinical carriers and four clinical cholera cases. A full-blown outbreak ensued that lasted through early May. During that period, Moore swabs in sewers were positive while increasing numbers of confirmed clinical cases and asymptomatic infections were detected. The acclimatization center was identified as the key source of infection of new workers (21). Positive Moore sewer swabs continued for 2 weeks after there were no further clinical or subclinical infections.

### Outbreaks of cholera in unexpected venues.

In the 1970s, sporadic cases of cholera gravis were confirmed in U.S. citizens who had not traveled. These U.S. cholera cases, which yielded an unusual highly hemolytic V. cholerae O1 El Tor Inaba strain, led to extensive use of Moore swabs to detect V. cholerae O1 in sewage, septic tanks, and environmental waters. Early in the 7th pandemic, El Tor biotype isolates typically were highly hemolytic, but by the 1970s, El Tor strains were uniformly nonhemolytic. Weissman et al. (22) isolated the same hemolytic V. cholerae O1 El Tor Inaba from a Moore swab placed in the septic tank draining the house of a 1973 Port La Vaca, Texas, patient as from the patient himself.

In 1978, 11 cases of cholera occurred along southwest Louisiana’s Gulf Coast, all due to hemolytic V. cholerae O1 El Tor Inaba. An epidemiologic investigation incriminated consumption of cooked crabs as the vehicle of transmission (90). Isolation of V. cholerae O1 from the municipal sewer system serving the town of the index case in Louisiana in 1978 indicated that there might be other cases (24). During September and October 1978, V. cholerae O1 El Tor Inaba was recovered from sewerage systems of six municipalities, while epidemiological investigations (including stool cultures) of persons with diarrheal illness in three of these towns confirmed cholera cases. Additionally, 11 of 21 Moore swabs from sewer systems serving communities where no cases of cholera were identified also grew V. cholerae O1.

V. cholerae O1 was detected in cultures of 7 of 10 (70%) Moore swabs placed in sewer lines serving homes of known infected persons prior to their receiving antibiotics, while the pathogen was isolated from only 1 of 16 (6%) swabs removed from sewers serving infected persons 1 to 7 days after they had commenced therapy with tetracycline (24). V. cholerae O1 was also recovered using Moore swabs from 2 of 4 septic tanks serving households of infected individuals. In one culture-negative septic tank, the Moore swabs had been placed 25 days after the patient was hospitalized; the other negative septic tank had “special treatment equipment.”

In one Louisiana town, Barrett et al. (24) used sewer swabs to track V. cholerae O1 in a manner reminiscent of Moore’s tracing the source of *S*. Paratyphi B. They recovered V. cholerae O1 from a Moore swab cultured on 9 September 1978 from the intake line of a sewage treatment plant. Four days thereafter, a second Moore swab was placed into the sewage treatment intake line, and swabs were also inserted into lines of 17 pumping stations draining different sections of town. Swabs from two sites yielded V. cholerae O1: one from the treatment plant intake and the second from a pumping station serving ∼50 blocks of dwellings. They then placed swabs in the tributary lines and, in 4 days, traced the source of infection to a 2-block area amenable to a Moore-like house-to-house search for the infected person(s). Meanwhile, a resident from this small area was admitted to the hospital with cholera gravis, and the stool culture grew V. cholerae O1. The patient’s daughter, who had subclinical V. cholerae O1 infection and remained at home, was the presumed source of V. cholerae O1 in the sewage line following her parent’s hospitalization (24).

With cholera, as with typhoidal *Salmonella*, Moore swabs can detect subclinical or mild infections of infected persons who do not seek medical care. Barrett et al. noted that in Louisiana (and most other jurisdictions), sewer swab surveillance was under the control of the local health department and did not require obtaining individual consent from the household members served by the sewers (24). Whereas sewer swab surveillance cannot detect infected persons not served by a sewerage system, septic tank surveillance is also effective, albeit more labor intensive (24). The CDC continues to recommend Moore swabs as a routine environmental surveillance method for the detection of V. cholerae O1 because of its practicality and effectiveness (91).

Two more isolated cases of cholera were reported in Texas in 1981. Moore swabs were placed in sewers of the three Texas cities harboring cases, and sewers draining houses of the cases yielded V. cholerae O1. These 1981 isolates were the same hemolytic El Tor Inaba strain exhibiting an identical restriction endonuclease pattern as the 1973 and 1978 Gulf of Mexico isolates, leading to the conclusion that this strain is indigenous to the environment of the Gulf of Mexico (25). It is now well established that V. cholerae O1 constitutes the autochthonous flora of brackish water environments where they adhere to chitinous zooplankton, whether in the Gangetic delta or the Gulf of Mexico.

### Epidemiologic investigation of an outbreak in a middle-income setting.

In July 1974, a small cluster of six cases of cholera due to V. cholerae O1 El Tor Ogawa occurred on the island of Guam, the U.S. Territory in the Mariana Islands (23). The cases occurred among construction workers who consumed an epidemiologically incriminated vehicle of transmission, a raw salted fish dish prepared from fish caught in Agana Bay. Moore swabs placed in the septic tank of one case and in the sewer downstream of the house of two other cases grew V. cholerae O1 El Tor Ogawa (23). V. cholerae O1 El Tor Ogawa was also isolated from two sewer lines that were not draining houses of known cholera patients and from water of the mouth of the Fonte River.

### Detection of live oral cholera vaccine in sewage.

In 1991, Simanjuntak et al. (26) investigated the safety, immunogenicity, and transmissibility of live oral cholera vaccine CVD 103-HgR compared to that of placebo in 200 children aged 2 to 5 years old in North Jakarta, Indonesia. After the administration of vaccine or placebo to the children, Moore swabs were placed in the open sewers or privies draining the latrines of 97 households of study participants to assess the environmental persistence of the vaccine strain. While non-O1 V. cholerae was recovered from human wastewater in 46 of the 97 sites, the CVD 103-HgR vaccine strain was not recovered from any specimens. These results support the sensitivity of the Moore swab method for detecting *Vibrio* species while establishing low transmissibility of the vaccine strain itself (26).

## DISCUSSION AND FUTURE DIRECTIONS

There are several fundamental challenges to sampling an urban environment for a pathogen that is transiently present (14, 18). Variables include unequivocal presence of a source of contamination, intermittent excretion, variability in pathogen load within the environmental source, diameter and flow rate of the sampling site, size of the population contributing fecal matter (household, block, neighborhood, district, and city), presence of disinfectants or antibiotics deleterious to the organism, variations in the materials used to prepare the swab and its dimensions, and variations in bacteriologic methods (Table 1).

**FIG 4:**
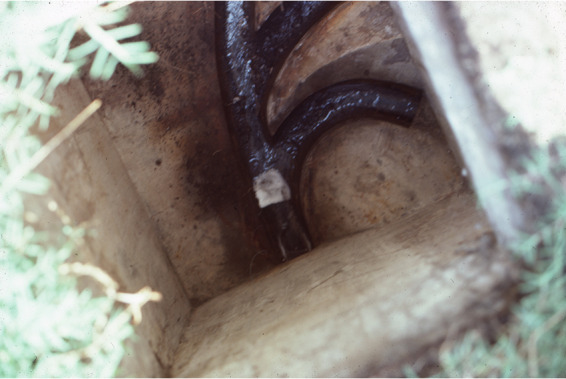
Sewer swab placed in the small diameter sewer draining three households in Santiago, Chile, in January 1983.

In Santiago, Chile, in 1983, Sears et al. (92) determined the sensitivity of the Moore swab under conditions designed to minimize these limitations: pipes draining the homes of bacteriologically confirmed asymptomatic chronic typhoid carriers (*n* = 10) were tested; each sewer was sampled for 48 to 72 h on 2 to 3 separate occasions during periods when the carriers were known to be home, usually Friday to Monday; small-diameter open sewer drains located on the household property that were shared with one or two other homes were tested (Fig. 4); and no carrier was taking antibiotics. Finally, standardized bacteriological methods were used to isolate *S*. Typhi from sewage (selenite-F broth enrichment; *Salmonella-Shigella* agar, Wilson and Blair [bismuth sulfite] agar, and deoxycholate citrate lactose sucrose agar to detect suspicious colonies; inoculation of suspect colonies into triple sugar iron agar slants; serotyping with specific antisera to confirm Typhi serovar; and phage typing of confirmed *S*. Typhi isolates). In total, 24 swabs were placed in sewers draining the homes of 10 known chronic carriers (minimum 2 swabs at different times per home). Six positive swabs correctly identified homes of 5 of the 10 carriers, indicating the method, as used, was 50% sensitive in identifying carrier households (92). Sears et al. (92) may have underestimated the sensitivity, since the period of sampling may not have in fact coincided with deposition of *S*. Typhi into the sewer by the carrier, particularly if shedding was intermittent or if the carrier did not use the connected toilet during the sampling period. In cases of institutional sewage surveillance (e.g., hospitals and prisons), disinfectants may diminish sensitivity of the culture-based bacteriology (93).

Efficacy of the Moore swab method to yield a viable pathogen also depends on the bacteriologic methods employed and the survival of the target organism. As pathogenic typhoidal *Salmonella* evolved into host-restricted serovars, traits that support enhanced survival in the environment were lost (94). Thus, organisms that have adapted to occupy environmental niches may out compete the human host-restricted pathogen in environmental samples.

*S*. Typhi may possibly exist in a “viable but nonculturable” (VBNC) state in water during adverse environmental conditions (95–97), as does V. cholerae O1 in brackish waters of the Gangetic delta. Under conditions of salinity, temperature, and pH of water during noncholera seasons, V. cholerae O1 remains alive but cannot be cultured directly; yet, it revives at the onset of cholera season when it becomes readily cultivable from environmental samples (98). Reversion to the cultivable state also occurs *in vivo* when VBNC V. cholerae O1 passes through the human intestine. This was documented in a clinical study where attenuated Vibrio cholerae O1 live oral vaccine strain CVD 101, when subjected to appropriate conditions, became noncultivable. However, an inoculum fed to healthy adult U.S. volunteers resulted in recovery of viable vaccine organisms from coprocultures from the volunteers (99). That VBNC nontyphoidal *Salmonella* does not regain infectivity in susceptible animals (100, 101) argues against the notion that VBNC typhoidal *Salmonella* regains infectivity when ingested by humans.

Nontyphoidal Salmonella enterica serovars survive and replicate inside intracellular vacuoles of waterborne free-living amoeba (*Acanthamoeba*) (102, 103). *S*. Typhi shows enhanced survival when incubated in the presence of Acanthamoeba castellanii (104), which may suggest that a similar symbiosis occurs in the environment. Such alternative environmental states of typhoidal *Salmonella*, if real, could be missed by the culture-based Moore swab.

Arguably, a true positive can also be defined as the presence of *S*. Typhi DNA in an environmental sample. Molecular methods, such as quantitative real-time PCR (qPCR), offer the potential to monitor drinking water for contamination with typhoidal *Salmonella* (105, 106) or to detect *S*. Typhi DNA in sewage samples from households of typhoid cases compared with those of controls (107). Ideally, a multitargeted PCR (108) and culture-based techniques should be combined to permit the isolation of viable organisms. *Salmonella* DNA persists (109), and molecular detection of bacteria from complex environmental matrices has notable challenges with both sensitivity and specificity at all stages of the process (108).

Bisha et al. (46) have adapted the filtration concept behind Moore’s sewer swab for a pump-based filtration device coined the “modified Moore swab (MMS),” in which cheese cloth or gauze is packed into a polyvinyl chloride pipe and a volume of water is pumped through the cloth. MMS is typically used to enumerate various fecal indicators and pathogens from surface water sources, including *Salmonella* spp., Escherichia coli, and Listeria monocytogenes, among others (42, 44, 46, 110–114). While quantitative, this modification of the Moore swab method has not yet been adapted to accommodate the intermittent excretion of *S*. Typhi and *S*. Paratyphi (115). MMS devices could prove useful for the filtration of typhoidal *Salmonella* if reconfigured to remain *in situ* for several days.

Recovery of a viable culturable organism is increasingly critical as disease surveillance and control efforts for typhoidal *Salmonella* shift toward using data from whole-genome sequencing (WGS), which requires a live organism (116). In these analyses, geospatial, geographical, and temporal data are integrated with WGS data derived from *S*. Typhi isolated from positive blood or stool cultures to monitor genotype circulation and antimicrobial resistance (117–120) and to assess *S*. Typhi population structures before and after a typhoid vaccination intervention (121). Few genomic epidemiology analyses apply WGS to environmentally derived typhoidal *Salmonella* isolates alongside clinical isolates. However, in one example, a genetic comparison completed in the mid-1990s using pulse-field gel electrophoresis (PFGE) to characterize clinical and environmental (sewage and river water) isolates from Santiago, Chile, from 1983 demonstrated overlap between the human and environmental samples (122). While the genomic resolution of PFGE is insufficient to decipher microcirculation patterns compared with that of WGS (123, 124), these data further supported the conclusion that irrigation of vegetable crops with untreated sewage was responsible for amplified long-cycle transmission that maintained typhoid endemicity in Santiago in the 1980s (69, 75, 76). Within this gap in typhoidal genomic epidemiology, the Moore swab may provide live culturable environmental isolates that, compared against human-derived isolates, will directly contribute to new models of transmission mechanics supporting either short-cycle or long-cycle transmission.

## CONCLUSION

This review details the practical utility of the Moore swab in outbreak and endemic settings for the isolation of viable *S*. Typhi, *S*. Paratyphi, and Vibrio cholerae O1 from various environmental sources, including sewage and surface waters. This tool may initially appear unappealing to the academic research community; however, when paired with quality-ensured diagnostic bacteriology, Moore’s technique has repeatedly exposed human reservoirs and environmental amplification mechanisms of these pathogens under varied circumstances. In several examples presented, the method’s flexibility proved critical to its public health utility. However, the challenges faced in sampling sewage and wastewater cannot be understated, and overcoming these should be a priority for funding agencies.

Modern advances in filtration technologies, molecular techniques, and next-generation sequencing have expanded and improved the precision of the toolkit available to public health laboratories, especially those located in high-income countries. Nevertheless, for low- and middle-income countries and for global academic researchers with limited funding, sustainable routine surveillance and bacteriology for enteric pathogens are essentially limited to affordable culture-based techniques. Indeed, Moore swabs in their classical applications have resurfaced in recent conference proceedings describing environmental surveillance programs for *S*. Typhi in Kolkata, India (125), and Samoa (126) and have been part of standardization efforts in the field (127, 128).

Given its simplicity and affordability, the Moore swab is well suited in resource-limited settings where typhoidal *Salmonella* is broadly endemic and sewage monitoring is feasible either through a municipal sewage network or household toilets connected to septic tanks. The identification of hot spots in a community or at certain public facilities (e.g., bus stations, rural hospitals, or ports and terminals) may inform epidemiological investigations, target vaccination campaigns, and help differentiate short-cycle versus long-cycle transmission patterns. PCR or advanced separation methods can improve the sensitivity of pathogen detection but should be combined with culture-based methods to recover viable organisms for further characterization. Subsequent *in vitro* studies may be performed, and next-generation genome sequencing can pave the way for genomic epidemiology and phylogeographical analyses connecting isolates from cases, contacts, and the environment.

Driven by the emergence and spread of *S*. Typhi harboring multiple antibiotic resistances (129) and availability of an efficacious new typhoid vaccine that is prequalified by the World Health Organization (130), the public health community is accelerating control of typhoid in many areas of endemicity (131, 132). Improved typhoidal disease surveillance and environmental microbiology can play important roles in guiding typhoid control, identifying human reservoirs, and incriminating specific modes of transmission that involve environmental contamination amenable to intervention. Where typhoid cases are rare, or in countries such as Fiji and Samoa, where mass vaccination efforts with typhoid vaccines are planned, sporadic and residual cases likely associated with human reservoirs of chronic biliary infection will become proportionately more important in the coming decade. These reservoirs and any associated environmental transmission pathways can be traced and identified through sewage networks, septic systems, and surface waters using Moore’s original application of the sewer swab.
